# Identification of a novel *LDLR* p.Glu179Met variant in Thai families with familial hypercholesterolemia and response to treatment with PCSK9 inhibitor

**DOI:** 10.1038/s41598-024-57069-z

**Published:** 2024-03-21

**Authors:** Burabha Pussadhamma, Chaiyasith Wongvipaporn, Atthakorn Wutthimanop, Manit Nuinoon, Sureerut Porntadavity, Nutjaree Jeenduang

**Affiliations:** 1 Department of Internal Medicine, Faculty of Medicine, Khon Kaen University, Khon Kaen, Thailand; 2 Queen Sirikit Heart Center of the Northeast, Khon Kaen University, Khon Kaen, Thailand; 3 Department of Internal Medicine, Maharaj Nakhon Si Thammarat Hospital, Nakhon Si Thammarat, Thailand; 4 School of Allied Health Sciences, Walailak University, Nakhon Si Thammarat, Thailand; 5 Faculty of Medical Technology, Mahidol University, Bangkok, Thailand

**Keywords:** Familial hypercholesterolemia, In silico analysis, *LDLR*, Novel variant, PCSK9 inhibitor, Genetics, Endocrinology

## Abstract

Familial hypercholesterolemia (FH) is a genetic disease characterized by elevated LDL-C levels. In this study, two FH probands and 9 family members from two families from northeastern Thailand were tested for *LDLR*, *APOB*, and *PCSK9* variants by whole-exome sequencing, PCR-HRM, and Sanger sequencing. In silico analysis of LDLR was performed to analyse its structure‒function relationship. A novel variant of *LDLR* (c.535_536delinsAT, p.Glu179Met) was detected in proband 1 and proband 2 in homozygous and heterozygous forms, respectively. A total of 6 of 9 family members were heterozygous for *LDLR* p.Glu179Met variant. Compared with proband 2, proband 1 had higher baseline TC and LDL-C levels and a poorer response to lipid-lowering therapy combined with a PCSK9 inhibitor. Multiple sequence alignment showed that LDLR p.Glu179Met was located in a fully conserved region. Homology modelling demonstrated that LDLR p.Glu179Met variant lost one H-bond and a negative charge. In conclusion, a novel *LDLR* p.Glu179Met variant was identified for the first time in Thai FH patients. This was also the first report of homozygous FH patient in Thailand. Our findings may expand the knowledge of FH-causing variants in Thai population, which is beneficial for cascade screening, genetic counselling, and FH management to prevent coronary artery disease.

## Introduction

Familial hypercholesterolemia (FH) is an autosomal dominant disorder^1^ characterized by high levels of low-density lipoprotein cholesterol (LDL-C) that subsequently leads to atherosclerosis and premature coronary artery disease (CAD)^1^. The clinical manifestations of FH are tendon xanthomas, xanthelasma, and corneal arcus^2^. FH results from variants in the low-density lipoprotein receptor (*LDLR*, OMIM 606945), apolipoprotein B (*APOB*, OMIM 107730), and proprotein convertase subtilisin/kexin type 9 (*PCSK9*, OMIM 607786) genes^3^. In addition, a rare autosomal recessive form of FH results from homozygous and compound heterozygous variants of the LDL receptor adapter protein 1 (*LDLRAP1*) gene^4^. Approximately 90% of FH patients with mutations have *LDLR* gene mutations^5^. Recently, more than 3000 *LDLR* variants have been reported in the ClinVar database. *APOB* and *PCSK9* mutations account for 6–10%^6^ and < 1%, respectively, of FH patients with mutations^7^. The frequency of homozygous FH (HoFH) is approximately 1:160,000–300,000^8,9^. In contrast, the frequency of heterozygous FH (HeFH) is approximately 1:250–300^8,9^.

Patients with homozygous FH have a more severe clinical phenotype than patients with heterozygous FH^10^. Moreover, the clinical FH phenotypes vary according to the type of *LDLR* mutation (receptor-negative or receptor-defective mutation), functional class, and residual LDLR activity^5,11^. The phenotypes are also influenced by other genetic and environmental factors^12^. FH is underdiagnosed and undertreated^9^. Early diagnosis and early treatment can prevent or delay the development of CAD^13^. In Thailand, research on FH is limited. Only two *LDLR* variants (p.Asp172Tyr and p.Met412Thr) have been reported in Thai FH patients^14,15^. In this study, we aimed to investigate gene variants in Thai FH patients and their family members.

## Materials and methods

### Subjects and families

Proband 1 and proband 2, who were unrelated CAD patients followed at the Srinagarind Hospital and Queen Sirikit Heart Center of the Northeast Region, Khon Kaen, Thailand, as well as 9 family members from their two families, were recruited for this study. Proband 1 and proband 2 were diagnosed with definite FH (DLCN score > 8 points) according to the Dutch Lipid Clinic Network (DLCN) criteria^16^. The DLCN scores of proband 1 and proband 2 were 22 and 10, respectively. Blood samples were collected from all subjects after they had fasted for 12 h. Total cholesterol, triglyceride, and  high-density lipoprotein cholesterol (HDL-C) levels were measured by enzymatic methods. LDL-C levels were calculated by the Friedewald equation^17^. The clinical follow-up data of the two probands were collected from medical records. The medication history of the family members of proband 1 and proband 2 was also investigated. The subjects provided informed consent before inclusion in the study. This study was approved by the Khon Kaen University Ethics Committee for Human Research (HE641105) and the Ethics Committee of Walailak University (WUEC-20-356-01). All methods in this study were performed in accordance with the relevant guidelines and regulations.

### Whole-exome sequencing (WES) and data analysis

The gene variant in proband 1 was identified by using WES. Genomic DNA was extracted from the buffy coat using a DNA extraction kit (Qiagen, Hilden, Germany). DNA quantification was performed using a Nanodrop ND-1000 spectrophotometer (NanoDrop Technologies, Wilmington, DE, USA). Genomic DNA was fragmented, and fragments of approximately 150–200 bp were then ligated to paired-end adaptors. Exome libraries and target enrichment were prepared using the SureSelect Human All Exon V5 + UTR-post Kit (Agilent Technologies, Santa Clara, CA). Exome sequencing was performed by an Illumina NovaSeq PE150 instrument (Illumina, San Diego, CA, USA). The average percentages of target coverage at sequencing depths of 1×, 10×, and 20× were 99.5%, 95%, and 86%, respectively. The data were mapped to the reference human genome hg19 from UCSC (original GRCh37 from NCBI, Feb. 2009) and analysed using BWA (Ver. Bwa-0.7.12), Picard (Ver. Picard-tools-1.130), GATK (Ver. GATKv3.4.0), and SnpEff (Ver. SnpEff_v4.1 g). The genetic variants were annotated using databases from 1000 Genomes Release Phase 3, dbSNP142, ClinVar release 05/2015, and the ESP (ESP6500SI_V2).

### PCR-HRM

*LDLR*, *APOB* (exon 26), and *PCSK9* (exon 7) gene variants were screened by PCR-HRM in proband 2^18^. A PCR-HRM reaction with a shift curve in melting temperature was utilized. PCR-HRM was performed using the Applied Biosystems QuantStudio 5 Real-Time PCR System (Applied Biosystems, USA). The reaction mixture included 2 μL of genomic DNA (10 ng/μL), 1 μL of forward and reverse primers (10 μM), 4 μL of 5 × HOT FIREPol® EvaGreen® HRM Mix (ROX) (Solis BioDyne, Germany) and 12 μL of water for a final volume of 20 μL. The primers and PCR-HRM conditions used for the detection of the *LDLR*, *APOB,* and *PCSK9* genes are listed in Tables S1, S2, and S3, respectively. HRM analysis was performed using High Resolution Melt Software v3.1 (Applied Biosystems, USA).

### Sanger sequencing

PCR and Sanger sequencing were performed to validate the *LDLR* p.Glu179Met variant in proband 1 and proband 2. All family members of proband 1 and proband 2 were also analysed for the *LDLR* p.Glu179Met variant by using PCR and Sanger sequencing. The primers and PCR conditions for the analysis of the *LDLR* p.Glu179Met variant (exon 4.3) are presented in Tables S1 and S4, respectively.

### Bioinformatics analysis

The Human Gene Mutation Database (HGMD) (https://www.hgmd.cf.ac.uk/ac/all.php), ClinVar (https://www.ncbi.nlm.nih.gov/clinvar), LOVD (https://databases.lovd.nl/shared/variants/LDLR), Exome Aggregation Consortium (ExAC) (http://exac.broadinstitute.org/), gnomAD (https://gnomad.broadinstitute.org/), and 1000 Genomes (http://www.1000genomes.org) databases were used to determine the novelty of the variant. In silico prediction of the effect of the *LDLR* variant was performed using SIFT (https://sift.bii.a-star.edu.sg/), PolyPhen-2 (http://genetics.bwh.harvard.edu/pph2/), and MutationTaster (http://www.mutationtaster.org/). The variant was classified according to the American College of Medical Genetics and Genomics (ACMG) guidelines^19^.

### Multiple sequence alignment

Multiple sequence alignment of LDLR sequences was performed using PRALINE software^20^. The alignment of LDLR sequences included the following species: *Homo sapiens* (P01130), *Macaca mulatta* (Q6S4M2), *Sus scrofa* (Q28832), *Oryctolagus cuniculus* (P20063), *Cricetulus griseus* (P35950), *Rattus norvegicus* (P35952), *Mus musculus* (P35951), *Gallus gallus* (Q7T2X3), *Xenopus laevis* (Q99087), *Chiloscyllium plagiosum* (P79708), and *Danio rerio* (Q7ZZT0), obtained from the Swiss-Prot database (http://www.expasy.ch/sprot/), and chimps (ENSPTRP00000017888), cows (ENSBTAT00000016342), and dogs (ENSCAFT00000027791), obtained from the Ensembl database (http://www.ensembl.org).

### Homology modelling

A model of the LDLR mutant protein was built using the crystal structure of the ligand binding domain and the LA3-LA4:RAP-D3 complex (PDB 2fcw, 1.26 Å) of LDLR as the template. The PDB files were retrieved from the Protein Data Bank (PDB) (http://www.rcsb.org/pdb/). Homology modelling was performed using Swiss-Pdb Viewer software^21^. The residues of receptor-associated protein (RAP) that bind to the ligand binding domain (LA3-LA4) of LDLR were selected and removed. The stereochemical quality of the model was verified using other validation programs, such as the WHATIF interface^22^, VERIFY3D^23^, and ERRAT^24^. Computations were conducted *in vacuo* with the GROMOS96 43B1 parameter set and implemented using the Swiss-Pdb Viewer^21^.

### Structural analysis

Swiss Pdb Viewer software^21^ was used to investigate the hydrogen bond patterns and the superimposition of the two wild-type and mutant protein models. The acidic and nonpolar residues were also assessed and coloured by the Swiss-Pdb Viewer^21^.

## Results

### Clinical characteristics of proband 1 and her family

**Figure 1:**
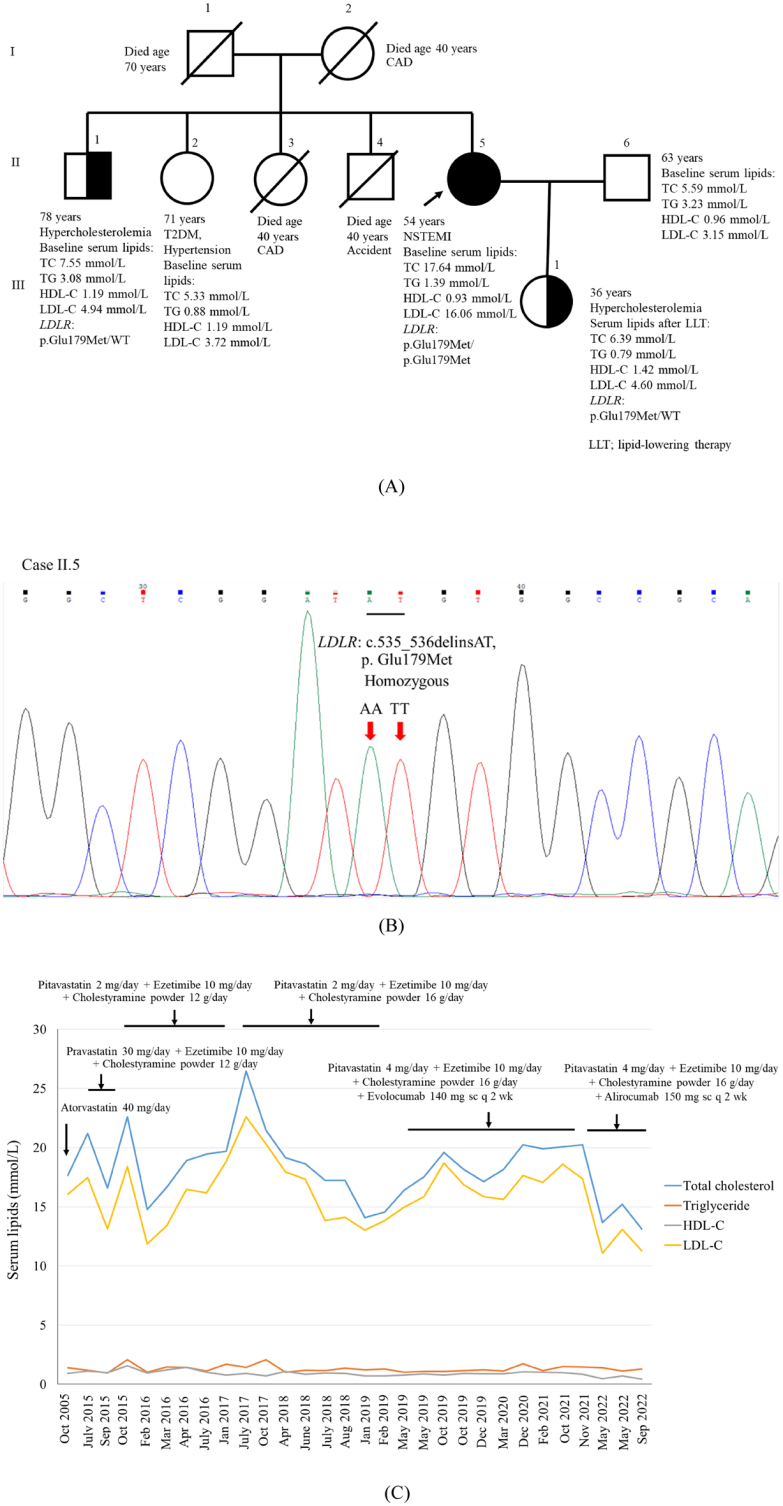
Pedigree of proband 1 (**A**) and DNA sequencing of proband 1 demonstrating homozygosity (**B**) of a novel variant of *LDLR*, c.535_536delinsAT, p.Glu179Met. Serum lipid levels of proband 1 during follow-up with LLT (**C**).

The clinical characteristics and pedigree of the family of proband 1 are shown in Table 1 and Fig. 1A, respectively. Proband 1 (II.5) had markedly elevated levels of total cholesterol (TC) and LDL-C at baseline. She presented with xanthelasma, corneal arcus, and tendon xanthoma on her elbows and feet. WES and Sanger DNA sequencing revealed that proband 1 was homozygous for the *LDLR* (c.535_536delinsAT; p.Glu179Met) variant (Fig. 1B). All *LDLR*, *APOB*, and *PCSK9* variants in proband 1 identified by WES is shown in Table S6. The father and mother of proband 1 died at the ages of 70 and 40 years, respectively. Two of the four siblings of proband 1 (II.3 and II.4) died due to a heart attack and an accident, respectively. Proband 1’s brother (II.1) and daughter (III.1) were positive for the heterozygous *LDLR* p.Glu179Met variant. Both had high levels of TC and LDL-C. However, the variant was not detected in proband 1’s sister (II.2) or her husband (II.6).

**Table 1:** Characteristics of the family of proband 1.

Variables	Proband 1’s family				
Subject	II.5	II.1	II.2	II.6	III.1
Relationship	Proband 1	Brother	Sister	Husband	Daughter
Gender (M/F)	F	M	F	M	F
Age of CAD diagnosis (years)	35	–	–	–	–
Current age (years)	54	78	71	63	36
Baseline total cholesterol (mmol/L)	17.64	7.55	5.33	5.59	6.39*
Baseline triglyceride (mmol/L)	1.39	3.08	0.88	3.23	0.79*
Baseline HDL-C (mmol/L)	0.93	1.19	1.19	0.96	1.42*
Baseline LDL-C (mmol/L)	16.06	4.94	3.72	3.15	4.60*
Sanger sequencing: *LDLR* p.Glu179Met variant	p.Glu179Met/p.Glu179Met	p.Glu179Met/WT	WT/WT	WT/WT	p.Glu179Met/WT
Clinical signs	Tendon xanthomas, Corneal arcus, Xanthelasma	–	–	–	–
Symptoms	Acute myocardial infarction, Hypercholesterolemia,	Hypercholesterolemia	T2DM, Hypertension	Normal	Hypercholesterolemia
Treatment	Pitavastatin 4 mg/day, Cholestyramine powder 16 g/day, Ezetimibe 10 mg/day, Alirocumab 150 mg every 2 weeks CABG (37 years of age), PCI (48, 49, and 55 years of age)	None	Anti-diabetic drug, anti-hypertensive drug	None	Pitavastatin 2 mg/day
Family history of CAD	Yes	Yes	Yes	Yes	Yes
Smoking	None	Ex-smoker	None	Ex-smoker	None

*Lipid profiles after lipid-lowering therapy.

### Clinical characteristics of proband 2 and her family

**Figure 2:**
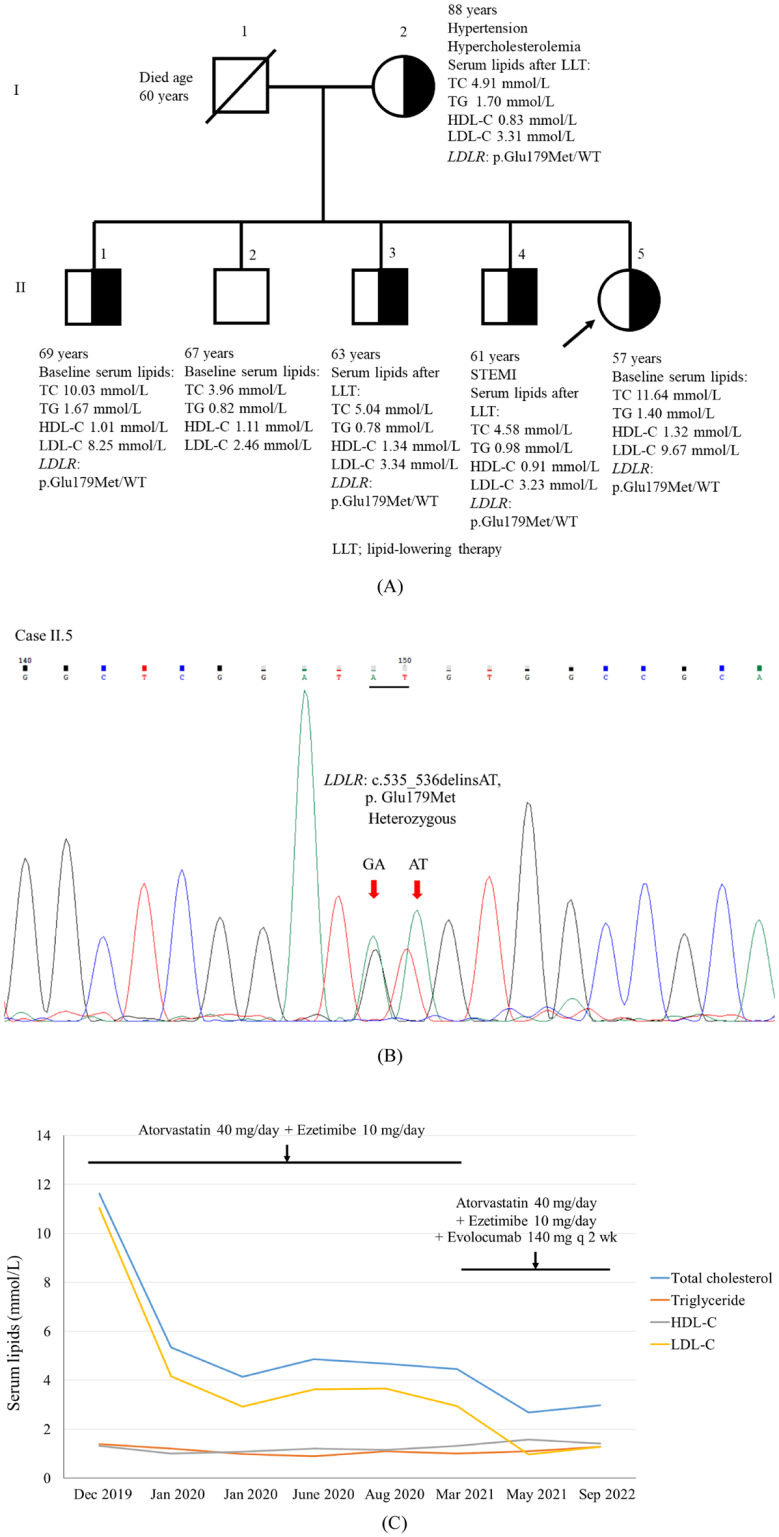
Pedigree of proband 2 (**A**) and DNA sequencing of proband 2 demonstrating heterozygosity (**B**) for a novel variant of *LDLR*, c.535_536delinsAT, p.Glu179Met. Serum lipid levels of proband 2 during follow-up with LLT (**C**).

The clinical characteristics and pedigree of the family of proband 2 are shown in Table 2 and Fig. 2A, respectively. Proband 2 (II.5) had high levels of TC and LDL-C. She developed unstable angina (UA) at 54 years of age. She had no tendon xanthomas, xanthelasma, or corneal arcus. Proband 2 was found to be heterozygous for the *LDLR* p.Glu179Met variant (Fig. 2B). *PCSK9* and *APOB* variants were not detected. The father of proband 2 died at 60 years of age. The mother of proband 2 and three of her four brothers (II.1, II.3, and II.4) were positive for the heterozygous *LDLR* p.Glu179Met variant (Table 2). All of them had high levels of TC and/or LDL-C. One of her brothers (II.4) developed CAD. The mother (I.2) and two brothers (II.3 and II.4) of proband 2 received lipid-lowering therapy (LLT). This variant was not detected in only one of her brothers (II.2).

**Table 2:** Characteristics of the family of proband 2.

Parameters	Proband 2’s family					
Subject	II.5	I.2	II.1	II.2	II.3	II.4
Relationship	Proband 2	Mother	Brother	Brother	Brother	Brother
Gender (M/F)	F	F	M	M	M	M
Age of CAD diagnosis (years)	54	–	–	–	–	57
Current age (years)	57	88	69	67	63	61
Baseline total cholesterol (mmol/L)	11.64	4.91*	10.03	3.96	5.04*	4.58*
Baseline triglyceride (mmol/L)	1.40	1.70*	1.67	0.82	0.78*	0.98*
Baseline HDL-C (mmol/L)	1.32	0.83*	1.01	1.11	1.34*	0.91*
Baseline LDL-C (mmol/L)	9.67	3.31*	8.25	2.46	3.34*	3.23*
Sanger sequencing: *LDLR* p.Glu179Met variant	p.Glu179Met/WT	p.Glu179Met/WT	p.Glu179Met/WT	WT/WT	p.Glu179Met/WT	p.Glu179Met/WT
Symptoms	Unstable angina, hypercholesterolemia	Hypercholesterolemia, hypertension	Hypercholesterolemia	Normal	Hypercholesterolemia, hypertension	STEMI, hypercholesterolemia, hypertension
Treatment	Atorvastatin 40 mg/day, ezetimibe 10 mg/day, evolocumab 140 mg every 2 weeks, CABG (55 years)	Atorvastatin 40 mg/day	None	None	Atorvastatin 40 mg/day	Atorvastatin 40 mg/day
Family history of CAD	Yes	Yes	Yes	Yes	Yes	Yes
Smoking	None	None	None	Ex-smoker	None	None

*Lipid profiles after lipid-lowering therapy.

### Phenotype variability of FH and the responsiveness to lipid-lowering therapy in proband 1 and proband 2

The 9-year clinical follow-up of proband 1 is shown in Fig. 1C. After receiving LLT with and without a proprotein convertase subtilisin/kexin type 9 inhibitor (PCSK9i), her LDL-C levels were reduced by 13.97 to − 24.65% and − 17.57%, respectively (Table S5). Despite the intensive LLT, her tendon xanthomas did not completely resolve. She underwent coronary artery bypass grafting (CABG) once at 37 years of age and percutaneous coronary intervention (PCI) 3 times at 48, 49, and 55 years of age. In comparison to proband 1, proband 2 had lower baseline serum lipid levels. The 4-year clinical follow-up of proband 2 is shown in Fig. 2C. After receiving LLT with and without PCSK9i, her LDL-C levels were reduced by − 89.79% and − 72.46%, respectively (Table S5). She underwent CABG at the age of 55 years.

### Bioinformatics analysis and multiple sequence alignment

**Figure 3:**
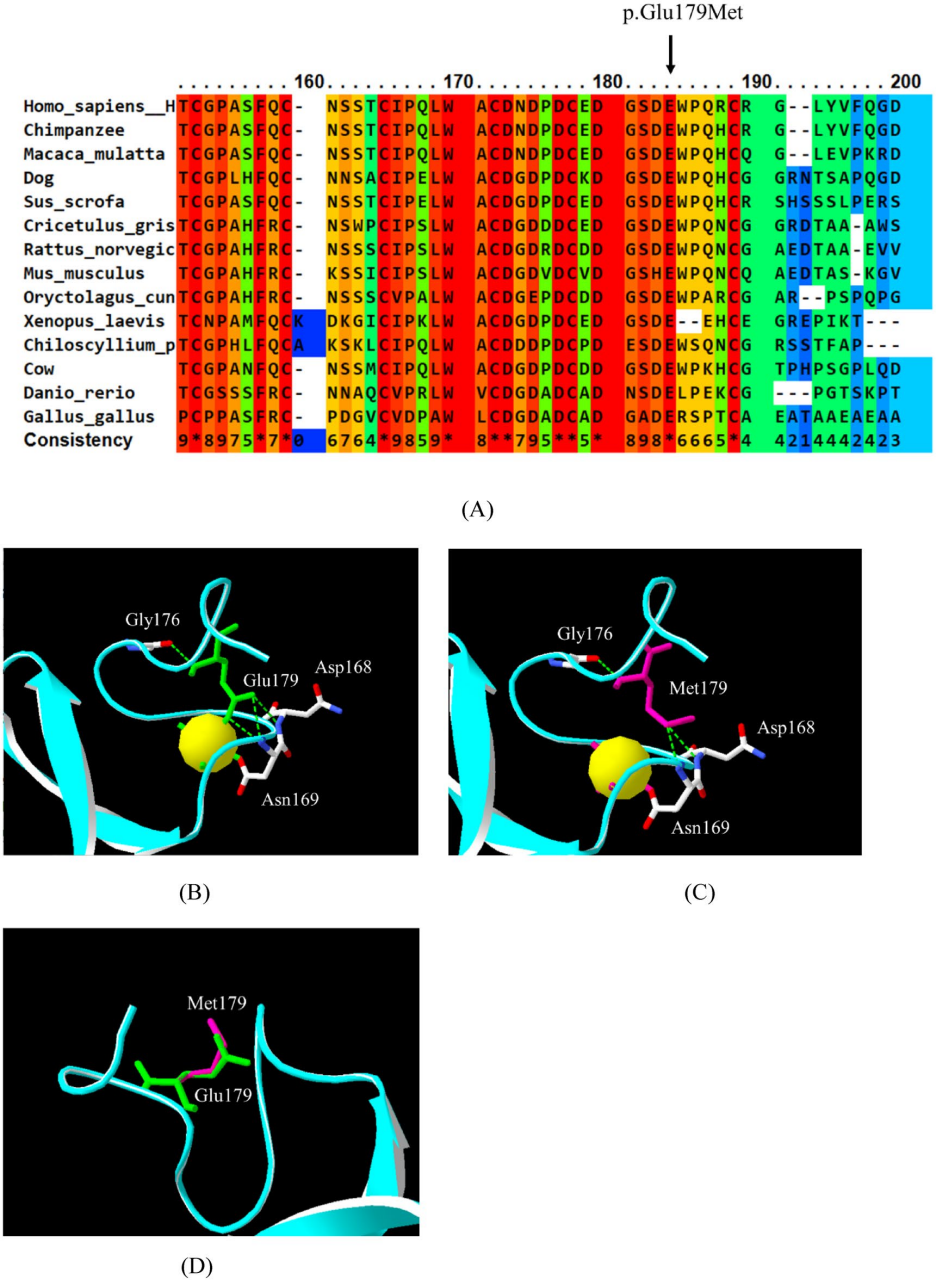

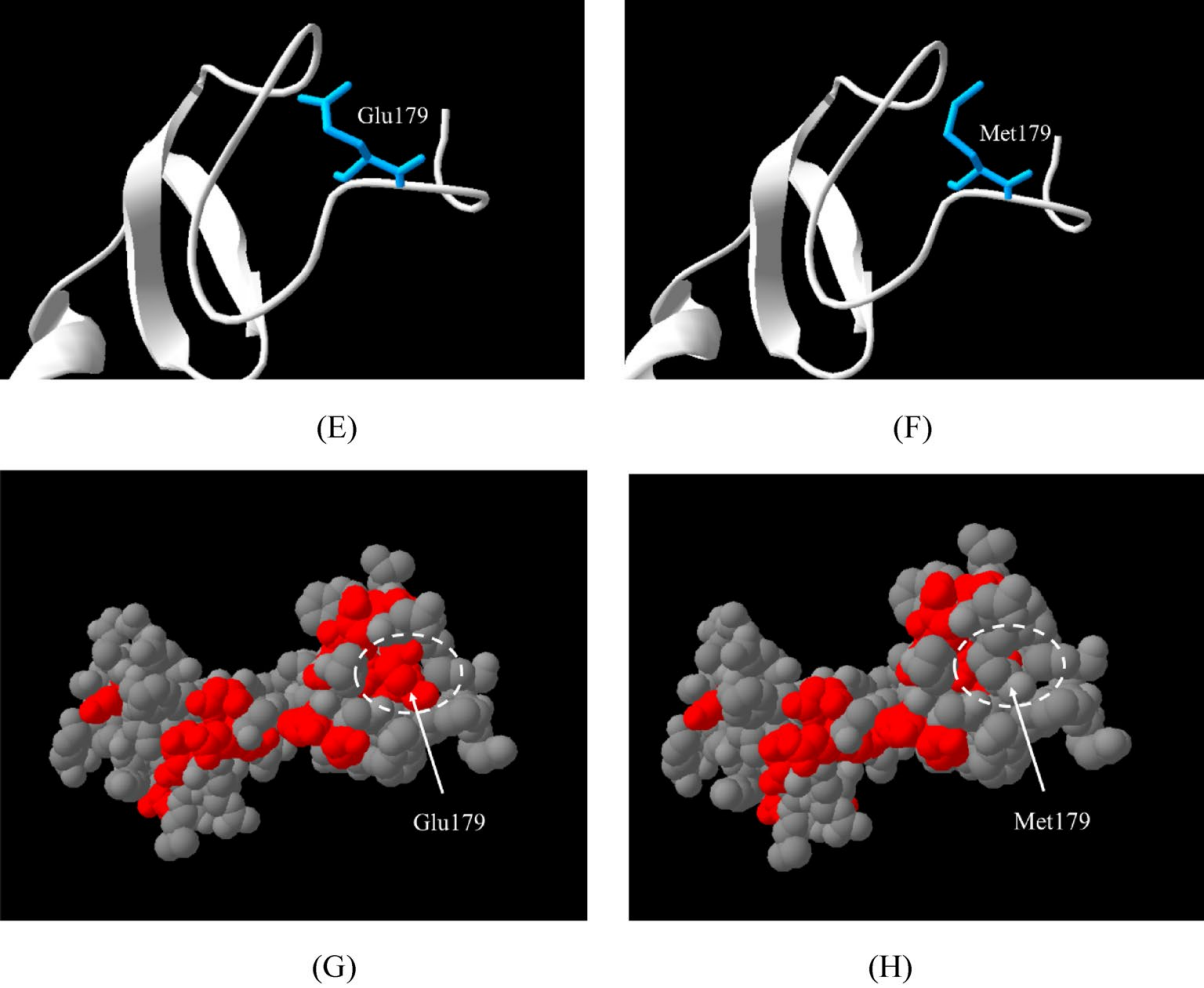
Multiple sequence alignment of LDLR proteins from several species (**A**). The position of the *LDLR* p.Glu179Met variant is indicated by an arrow. The conserved score ranged from 0 for the least conserved alignment position to 10 (*) for the most conserved alignment position. Homology modelling of the LDLR p.Glu179Met variant (B-H). The ribbon structures show the hydrogen bond patterns of the wild type (**B**) and p.Glu179Met (**C**) models. The amino acid side chains of Glu179 and Met179 are shown in green and pink, respectively. Green dotted lines: hydrogen bonds. Yellow spheres: calcium ions. Superimposition of the Glu179 model (green) and the Met179 model (pink) (**D**). The structures of the Glu179 model (**E**) and Met179 model (**F**) are coloured according to the accessible surface area (ASA). Dark blue represents completely buried residues, whereas light blue, green, orange and yellow represent residues with increasing accessible surface areas. The negatively charged residue is coloured red for the Glu179 model (**G**). Nonpolar residues are coloured grey for the Met179 model (**H**).

SIFT, PolyPhen-2, and MutationTaster predicted the LDLR p.Glu179Met variant to be deleterious (0.000), probably damaging (0.999), and disease causing (0.9999), respectively. The multiple sequence alignment for LDLR is shown in Fig. 3. The LDLR p.Glu179Met variant is located at a fully conserved residue (conserved score = 10) and in a conserved acidic amino acid region (**D**XXX**D**XX**D**XX**DE;** acidic residues are shown in bold, and X is any amino acid) of LDLR. The *LDLR* p.Glu179Met variant was not found in the HGMD, ClinVar, LOVD, ExAC, gnomAD, or 1000 Genomes databases. According to ACMG guidelines, the novel *LDLR* p.Glu179Met variant was classified as likely pathogenic because it met 2 moderate (PM1 and PM2) and 4 supporting (PP1, PP3, PP4, and PS4) criteria (Table S7).

### Homology modelling

To investigate the structure and function of the LDLR p.Glu179Met variant, an in silico homology modelling analysis was performed. LDLR p.Glu179Met is located in the 4th repeat (LA4) module of the ligand binding domain (LBD) of LDLR. Homology modelling of LDLR p.Glu179Met showed changes in the hydrogen bond pattern. In the wild-type model, the Glu179 residue formed four hydrogen bonds with its structural neighbours (Gly176, Asp168, and Asn169 residues) (Fig. 3B). In contrast, the mutant model of LDLR Met179 formed three hydrogen bonds among the Gly176, Asp168, and Asn169 residues (Fig. 3C). This substitution altered the side chain from a polar hydrophilic charge with Glu179 to an aliphatic hydrophobic and neutral charge with the Met179 residue. Thus, this may result in the loss of a negative charge at position 179. This loss is shown in Fig. 3G and H by the difference in colour due to the negative charge of the residue. However, after the wild-type and mutant models were superimposed, the main chain structure did not change significantly (Fig. 3D). The solvent accessibility surface areas (ASAs) of the Glu179 and Met179 residues were not different (Fig. 3E and F).

## Discussion

To our knowledge, this is the first study to report a novel *LDLR* p.Glu179Met variant in two families of FH patients from northeastern Thailand. Proband 1, who was homozygous for the novel *LDLR* p.Glu179Met variant, exhibited a severe FH phenotype with massive tendon xanthomas, corneal arcus, premature CAD, and a poor response to LLT. Although proband 1 received intensive LLT (e.g., statins, ezetimibe, and PCSK9i), her serum LDL-C levels did not reach the LDL-C target. Moreover, her tendon xanthomas did not completely resolve. A previous study also demonstrated that the size of tendon xanthomas in some FH patients did not decrease after intensive LLT (e.g., statins plus PCSK9i)^25^. This may be due to the severity of hypercholesterolemia, the chronic nature of tendon xanthomas, and the amount of fibrous tissue in tendon xanthomas. However, proband 2, who was heterozygous for the novel *LDLR* p.Glu179Met variant, did not present any clinical signs. Compared with proband 1, proband 2 seemed to have a less severe FH phenotype, including lower baseline serum lipid levels and a better response to LLT (e.g., statins combined with PCSK9i). HoFH patients typically have a more severe FH phenotype than HeFH patients^26^.

Similar to proband 1, some HoFH patients in Mexico^27^, India^28^, and China^29^ who were treated with PCSK9i did not achieve the LDL-C goal (< 50% LDL-C reduction from baseline)^26^. This suggested that the residual LDLR activity in these HoFH patients was minimal or absent. However, in an African American woman with HoFH who received a combination of atorvastatin, ezetimibe, PCSK9i (alirocumab), and evinacumab, a novel monoclonal antibody against angiopoietin-like 3 (ANGPTL3), LDL-C decreased to < 1.81 mmol/L^30^. Evinacumab has been found to reduce LDL-C by 50%, independent of LDLR activity^31^. Therefore, other drugs, such as ANGPTL3 inhibitors and lomitapide, which reduce LDL-C levels in a manner independent of LDLR activity, should be considered for HoFH patients with poorer responses^32^.

In this study, proband 2 and six family members carried the heterozygous *LDLR* p.Glu179Met variant. None of them presented clinical signs; however, one patient developed premature CAD. The patients’ baseline serum LDL-C levels varied from 4.94 to 9.67 mmol/L. A wide spectrum of LDL-C levels (≥ 3.36 mmol/L to approximately 12.93 mmol/L) among HeFH patients has been reported previously^33^. Studies have suggested that phenotypic variability among HeFH patients may result from genetic background and environmental factors, e.g., a Westernized lifestyle, dietary intake, exercise, menopause, PCSK9 levels, and smoking^12,34^.

In the present study, a novel delins (c.535_536delinsAT) variant of the *LDLR* gene resulted in nonconserved substitution of glutamic acid with methionine at position 179 of the 4th repeat of the ligand binding domain (LBD) of LDLR. This variant is located in the DXXXDXXDXXDE sequence, which is a fully conserved acidic sequence^35^. This sequence is involved in calcium coordination, which is essential for maintaining the active conformation^36^ and binding apoB-100 and apoE-containing lipoproteins^37^. According to the HGMD, ClinVar, gnomAD, and LOVD databases, other *LDLR* variants at the same position, e.g., p.Glu179Lys, p.Glu179X, and p.Glu179Gly, have also been reported to cause FH in Italy, France, and Norway, respectively. In addition, other variants in the LBD region of LDLR that caused the substitution of glutamic acid with other residues, e.g., p.Glu101Lys, p.Glu140Lys, p.Glu208Lys, p.Glu228Lys, p.Glu228Gln, and p.Glu240Lys, decreased LDLR activity to 15–30%, 30%, 5–15%, < 2%, 2–5%, and 15–30%, respectively. These variants were classified as class II variants or transport defects^38^. Some *LDLR* variants in the LBD region of LDLR, e.g., p.Asp224Gly, p.Asp227Glu, p.Glu228Lys, p.Glu240Lys and p.Asp266Glu, also led to protein folding defects^39,40^.

Overall, we hypothesized that the *LDLR* p.Glu179Met variant, which lacks one H bond and a negative charge, may disturb calcium coordination and may further lead to a conformational change. The misfolding of the protein structure and the loss of the negative charge of glutamic acid in the *LDLR* p.Glu179Met variant may interrupt binding to apoB-100 and apoE-containing lipoproteins and may result in decreased residual LDLR activity. This might be the reason that the HoFH patient in this study was resistant to intensive LLT. In this study, because the two probands lived in nearby districts in northeastern Thailand, we suggest that the *LDLR* p.Glu179Met variant may be a common or founder variant among this population. The epidemiology of the *LDLR* p.Glu179Met variant should be further investigated in these areas. A major limitation of this study is that a functional analysis of the *LDLR* p.Glu179Met variant was not performed. In addition, large deletions and insertions in *LDLR* were not analysed. In conclusion, a novel *LDLR* p.Glu179Met variant was identified for the first time in Thai FH patients. This was also the first report of a HoFH patient in Thailand. Our findings may expand the knowledge of FH-causing variants in the Thai population, which is beneficial for cascade screening, genetic counselling, and FH management to prevent CAD.

### Supplementary Information

Supplementary Tables.

## Data Availability

All the data generated or analysed during this study are included in this published article. The datasets generated and/or analysed during the current study are available in the ClinVar database (https://www.ncbi.nlm.nih.gov/clinvar/variation/2672294/) under accession number SCV004176840.
