# Supplementary material for: Identification of a novel LDLR p.Glu179Met variant in Thai families with familial hypercholesterolemia and response to treatment with PCSK9 inhibitor
Source: Sci Rep. 2024 Mar 21;14:6785. doi: 10.1038/s41598-024-57069-z (PMC10957951; doi:10.1038/s41598-024-57069-z)
Supplement: Supplementary file 1 — Supplementary Tables. [file 41598_2024_57069_MOESM1_ESM.docx]

Table S1 Lists of primers for PCR-HRM analysis of *LDLR*, *APOB*, and *PCSK9* genes.

| No. | Name | Forward primer (5’-3’) | Name | Reverse primer (5’-3’) |
| --- | --- | --- | --- | --- |
| 1 | FP promoter LDLR | CAGCTCTTCACCGGAGACCC | RP promoter LDLR | ACCTGCTGTGTCCTAGCTGG |
| 2 | FP exon 1 LDLR | ACTCCTCCCCCTGCTAGAAACCTCA | RP exon 1 LDLR | CTATTCTGGCGCCTGGAGCAAGCC |
| 3 | FP exon 2 LDLR | TTGAGAGACCCTTTCTCCTTTTCC | RP exon 2 LDLR | GCATATCATGCCCAAAGGGG |
| 4 | FP exon 3 LDLR | TCAGTGGGTCTTTCCTTTGAG | RP exon 3 LDLR | CAGGACCCCGTAGAGACAAA |
| 5 | FP exon 4(1) LDLR | TGGTGTTGGGAGACTTCACA | RP exon 4(1) LDLR | CACTCATCCGAGCCATCTTC |
| 6 | FP exon 4(2) LDLR | AAGTGCATCTCTCGGCAGTT | RP exon 4(2) LDLR | CCCCTTGGAACACGTAAAGA |
| 7 | FP exon 4(3) LDLR | AGCTTCCAGTGCAACAGCTC | RP exon 4(3) LDLR | CATACCGCAGTTTTCCTCGT |
| 8 | FP exon 4(4) LDLR | TGTTCCAAGGGGACAGTAGC | RP exon 4(4) LDLR | AAATCACTGCATGTCCCACA |
| 9 | FP exon 5 LDLR | AGAAAATCAACACACTCTGTCCTG | RP exon 5 LDLR | GGAAAACCAGATGGCCAGCG |
| 10 | FP exon 6 LDLR | TCCTCCTTCCTCTCTCTGGC | RP exon 6 LDLR | TCTGCAAGCCGCCTGCACCG |
| 11 | FP exon 7 LDLR | GGCGAAGGGATGGGTAGGGG | RP exon 7 LDLR | GTTGCCATGTCAGGAAGCGC |
| 12 | FP exon 8 LDLR | CATTGGGGAAGAGCCTCCCC | RP exon 8 LDLR | GCCTGCAAGGGGTGAGGCCG |
| 13 | FP exon 9 LDLR | TCCATCGACGGGTCCCCTCTGACCC | RP exon 9 LDLR | AGCCCTCATCTCACCTGCGGGCCAA |
| 14 | FP exon 10(1) LDLR | AGATGAGGGCTCCTGGCGCTGATGCC | RP exon 10(1) LDLR | GCCCTTGGTATCCGCAACAGAGACA |
| 15 | FP exon 10(2) LDLR | GATCCACAGCAACATCTACTGGACC | RP exon 10(2) LDLR | AGCCCTCAGCGTCGTGGATA |
| 16 | FP exon 11 LDLR | TCCTCCCCCGCCCTCCAGCC | RP exon 11 LDLR | GCTGGGACGGCTGTCCTGCG |
| 17 | FP exon 12 LDLR | GCACGTGACCTCTCCTTATCCACTT | RP exon 12 LDLR | CACCTAAGTGCTTCGATCTCGTACG |
| 18 | FP exon 13 LDLR | GTCATCTTCCTTGCTGCCTG | RP exon 13 LDLR | GTTTCCACAAGGAGGTTTCAAGGTT |
| 19 | FP exon 14 LDLR | GAATCTTCTGGTATAGCTGAT | RP exon 14 LDLR | GCAGAGAGAGGCTCAGGAGG |
| 20 | FP exon 15 LDLR | GGCACGTGGCACTCAGAAGAC | RP exon 15 LDLR | ACCCGTCTCTGGGTGAAGAGG |
| 21 | FP exon 16 LDLR | CCTTCCTTTAGACCTGGGCC | RP exon 16 LDLR | CATAGCGGGAGGCTGTGACC |
| 22 | FP exon 17 LDLR | GGGTCTCTGGTCTCGGGCGC | RP exon 17 LDLR | GGCTCTGGCTTTCTAGAGAGGG |
| 23 | FP exon 18 LDLR | GCCGTGTTTCCTGAATGCTGG | RP exon 18 LDLR | TCTCAGGAAGGGTTCTGGGC |
| 24 | FP exon 26 APOB | TGTCAAGGGTTCGGTTCTTT | RP exon 26 APOB | GGGTGGCTTTGCTTGTATGT |
| 25 | FP exon 7 PCSK9 | CCCTCTCTTGGGCTCCTTTCT | RP exon 7 PCSK9 | AAAGGGGCTGTTAGCATCACG |

Table S2 PCR-HRM conditions for the analysis of *LDLR*, *APOB* (exon 26), and *PCSK9* (exon 7) gene variants.

| Mutations | Stage | Step | Temperature (°C) | Time | Cycle |
| --- | --- | --- | --- | --- | --- |
| *LDLR*,  *APOB* (exon 26),  and *PCSK9* (exon 7) | Hold stage | Step 1 | 95 | 15 min | 1 |
|  | PCR stage | Denaturation | 95 | 15 s | 40 |
|  |  | Annealing | *** | 20 s |  |
|  |  | Extension | 72 | 20 s |  |
|  | Melt curve stage | Step 1 | 95 | 15 s | 1 |
|  |  | Step 2 | 60 | 1 min |  |
|  |  | Step 3 | 98 | 15 s |  |

***Annealing temperature of each fragment is shown in Table S3.

Table S3 Tm of each fragment for PCR-HRM.

| No. | Fragment | Gene | Annealing temperature (^o^C) |
| --- | --- | --- | --- |
| 1 | 4 | LDLR exon 3 | 57 |
| 2 | 20 | LDLR exon 15 |  |
| 3 | 18 | LDLR exon 13 | 61 |
| 4 | 23 | LDLR exon 18 |  |
| 5 | 1 | LDLR promoter | 62 |
| 6 | 3 | LDLR exon 2 |  |
| 7 | 5 | LDLR exon 4.1 |  |
| 8 | 6 | LDLR exon 4.2 |  |
| 9 | 7 | LDLR exon 4.3 |  |
| 10 | 9 | LDLR exon 5 |  |
| 11 | 10 | LDLR exon 6 |  |
| 12 | 15 | LDLR exon 10.2 |  |
| 13 | 17 | LDLR exon 12 |  |
| 14 | 19 | LDLR exon 14 |  |
| 15 | 24 | APOB exon 26 |  |
| 16 | 25 | PCSK9 exon 7 |  |
| 17 | 2 | LDLR exon 1 | 67 |
| 18 | 11 | LDLR exon 7 |  |
| 19 | 13 | LDLR exon 9 |  |
| 20 | 14 | LDLR exon 10.1 |  |
| 21 | 21 | LDLR exon 16 |  |
| 22 | 22 | LDLR exon 17 |  |
| 23 | 12 | LDLR exon 8 | 71 |
| 24 | 16 | LDLR exon 11 |  |
| 25 | 8* | LDLR exon 4.4 | * |

*Fragment 8 could not be analysed by PCR-HRM. The fragment was screened for mutations by using DNA sequencing.

Table S4 PCR conditions for analysis of the *LDLR* (exon 4.3) gene.

| Allele | Step | Temperature (℃) | Time | Cycle |
| --- | --- | --- | --- | --- |
| *LDLR* (exon 4.3) | Pre-denaturation | 95 | 5 min | 1 |
|  | Denaturation | 95 | 30 s | 40 |
|  | Annealing | 60 | 30 s |  |
|  | Extension | 72 | 30 s |  |
|  | Final extension | 72 | 5 min | 1 |

Table S5 Effect of lipid-lowering therapy (LLT) on the serum lipid profiles of proband 1 and proband 2.

|  | Baseline | After the combination of LLT without PCSK9i  (Group A) | After the combination of LLT with evolocumab  (Group B) | After the combination of LLT with  alirocumab  (Group C) | % reduction  (baseline vs. Group A) | % reduction  (baseline vs. Group B) | % reduction  (baseline vs. Group C) |
| --- | --- | --- | --- | --- | --- | --- | --- |
| Proband 1 | | | | | | | |
| TC (mmol/l) | 17.64 | 14.56 | 19.89 | 13.14 | -17.45% | 12.76% | -25.51% |
| TG (mmol/l) | 1.39 | 1.29 | 1.17 | 1.29 | -7.32% | -15.45% | -7.31% |
| HDL-C (mmol/l) | 0.93 | 0.72 | 1.03 | 0.44 | -22.22% | 11.11% | -52.78% |
| LDL-C (mmol/l) | 16.06 | 13.24 | 18.31 | 12.10 | -17.57% | 13.97% | -24.65% |
| Proband 2 | | | | | | | |
| TC (mmol/l) | 11.64 | 4.45 | 2.99 | NA | -61.78% | -74.22% | NA |
| TG (mmol/l) | 1.40 | 1.00 | 1.28 | NA | -28.23% | -8.87% | NA |
| HDL-C (mmol/l) | 1.32 | 1.32 | 1.42 | NA | 0% | 7.84% | NA |
| LDL-C (mmol/l) | 9.67 | 2.66 | 0.98 | NA | -72.46% | -89.79% | NA |

NA; not applicable

Table S6 All *LDLR, APOB*, and *PCSK9* variants in proband 1 identified by whole-exome sequencing (WES).

| Chromosome | Zygosity | Gene | Location | Genetic variant | Protein variants | dbSNP142_  ID |
| --- | --- | --- | --- | --- | --- | --- |
| 19 | HOM | *LDLR* | Exon 4 | c.535G>A, c.536A>T | p.Glu179Met | - |
| 19 | HOM | *LDLR* | Intron 6 | c.940+36G>A | - | rs13306513 |
| 19 | HOM | *LDLR* | Intron 7 | c.1060+7T>C | - | rs2738442 |
| 19 | HOM | *LDLR* | Exon 10 | c.1413A>G | p.Arg471Arg | rs5930 |
| 19 | HOM | *LDLR* | Exon 11 | c.1617C>T | p.Pro539Pro | rs5929 |
| 19 | HOM | *LDLR* | Intron 11 | c.1705+56C>T | - | rs4508523 |
| 19 | HOM | *LDLR* | Intron 11 | c.1706-69G>T | - | rs7259278 |
| 19 | HOM | *LDLR* | Intron 11 | c.1706-55A>C | - | rs2738447 |
| 19 | HOM | *LDLR* | Intron 12 | c.1845+141_1845+142delTT | - | - |
| 19 | HOM | *LDLR* | Exon 15 | c.2232A>G | p.Arg744Arg | rs5927 |
| 19 | HOM | *LDLR* | Intron 15 | c.2312-136A>G | - | rs2569538 |
| 19 | HOM | *LDLR* | Exon 18 | c.*2210_*2211  delTA | - | rs142742959 |
| 2 | HET | *APOB* | Exon 29 | c.13013G>A | p.Ser4338Asn | rs1042034 |
| 2 | HET | *APOB* | Exon 26 | c.8216C>T | p.Pro2739Leu | rs676210 |
| 2 | HET | *APOB* | Exon 26 | c.7545C>T | p.Thr2515Thr | rs693 |
| 2 | HOM | *APOB* | Exon 26 | c.6937A>G | p.Ile2313Val | rs584542 |
| 2 | HET | *APOB* | Exon 26 | c.6936C>T | p.Asp2312Asp | rs1041968 |
| 2 | HOM | *APOB* | Exon 26 | c.4265A>G | p.Tyr1422Cys | rs568413 |
| 2 | HET | *APOB* | Exon 25 | c.4163G>A | p.Arg1388His | rs13306187 |
| 2 | HOM | *APOB* | Exon 24 | c.3843-77T>C | . | rs488329 |
| 2 | HET | *APOB* | Exon 23 | c.3697-79C>T | . | rs673548 |
| 2 | HET | *APOB* | Exon 14 | c.1853C>T | p.Ala618Val | rs679899 |
| 2 | HET | *APOB* | Exon 13 | c.1626G>T | p.Glu542Asp | rs367788462 |
| 2 | HET | *APOB* | Intron 12 | c.1617+142_1617+145delTCCT | . | rs533954660 |
| 2 | HOM | *APOB* | Intron 9 | c.1125-121_1125-120insAGATG | . | rs10701091 |
| 2 | HET | *APOB* | Exon 6 | c.581C>T | p.Thr194Met | rs13306198 |
| 2 | HET | *APOB* | Exon 4 | c.293C>T | p.Thr98Ile | rs1367117 |
| 1 | HOM | *PCSK9* | Intron 1 | c.207+15A>G | . | rs2495482 |
| 1 | HOM | *PCSK9* | Intron 3 | c.524-1170G>T | . | rs599037 |
| 1 | HET | *PCSK9* | Intron 3 | c.524-90C>G | . | rs613855 |
| 1 | HET | *PCSK9* | Intron 3 | c.524-68C>G | . | rs624612 |
| 1 | HET | *PCSK9* | Intron 4 | c.657+82G>A | . | rs625619 |
| 1 | HET | *PCSK9* | Intron 4 | c.657+107delC | . | rs397735050 |
| 1 | HET | *PCSK9* | Intron 4 | c.658-7C>T | . | rs2483205 |
| 1 | HET | *PCSK9* | Intron 5 | c.799+3A>G | . | rs2495477 |
| 1 | HET | *PCSK9* | Intron 5 | c.799+64C>A | . | rs494198 |
| 1 | HOM | *PCSK9* | Exon 7 | c.1026A>G | p.Gln342Gln | rs509504 |
| 1 | HOM | *PCSK9* | Intron 8 | c.1355-56C>T | . | rs585131 |
| 1 | HOM | *PCSK9* | Exon 9 | c.1380A>G | p.Val460Val | rs540796 |
| 1 | HOM | *PCSK9* | Exon 9 | c.1420G>A | p.Val474Ile | rs562556 |
| 1 | HOM | *PCSK9* | Intron 10 | c.1681+64G>A | . | rs483462 |
| 1 | HOM | *PCSK9* | Intron 11 | c.1863+94G>A | . | rs630431 |
| 1 | HOM | *PCSK9* | Exon 12 | c.2009G>A | p.Gly670Glu | rs505151 |
| 1 | HOM | *PCSK9* | Exon 12 | c.*571C>T | . | rs662145 |
| 1 | HOM | *PCSK9* | . | c.*2441T>G | . | rs560784 |

HOM; Variant is homozygous, HET; Variant is heterozygous

Table S7 Pathogenicity of the *LDLR* p.Glu179Met variant classified by the ACMG criteria.

|  | Description | Evidence |
| --- | --- | --- |
| Moderate criteria | | |
| PM1 | Missense variant is located in exon 4 | *LDLR* p.Glu179Met variant is located in exon 4. |
| PM2 | Variant has a PopMax MAF <0.0002 (0.02%) in gnomAD. | *LDLR* p.Glu179Met variant is absent from gnomAD. |
| Supporting criteria | | |
| PP1 | Variant segregates with phenotype in 2-3 informative meioses in ≥1 family. Must include ≥1 affected relative (LDL-C >75^th^ percentile) with the variant. | *LDLR* p.Glu179Met variant segregates with phenotype in 2 families and ≥1 affected relative (LDL-C >75^th^ percentile) carries the variant. |
| PP3 | Computational evidence supports a deleterious effect on the gene or gene product. | *LDLR* p.Glu179Met variant loses one H bond and a negative charge according to *in silico* analysis. |
| PP4 | Any *LDLR* variant identified in an FH patient [diagnosis based on any validated clinical criteria, ex. Dutch Lipid Clinic Network (≥6), Simon Broome (possible/definite), MEDPED], after alternative causes of high cholesterol are excluded. | Proband 1 and proband 2 carrying the *LDLR* p.Glu179Met variant were diagnosed with definite FH according to a Dutch Lipid Clinic Network (DLCN) score ≥ 6.  The DLCN scores of proband 1 and proband 2 were 22 and 10, respectively. |
| PS4 | Variant is found in 2-5 unrelated FH cases (FH diagnosis made by any validated clinical criteria). | Variant is found in 2 unrelated FH cases (FH diagnosis made by DLCN criteria). |
|  | Final classification | Likely pathogenic;  2 moderate, and 4 supporting criteria |
